# Parietal peritoneal lipomas: a first case report of two lipomas of the parietal peritoneum

**DOI:** 10.1093/jscr/rjab162

**Published:** 2021-05-04

**Authors:** Yagan Pillay

**Affiliations:** Department of General Surgery, University of Saskatchewan, Saskatchewan, Canada

## Abstract

Parietal peritoneal lipomas are a rare surgical entity with seven case reports in the published literature. Their aetiology remains nebulous and includes theories such as misplaced embryonic tissue, adipose hyperproliferation, trauma and fat herniation or excessive obesity. This is the first case report in the literature with two parietal peritoneal lipomas incarcerated in an umbilical hernia. We strongly advocate for an international rare tumour registry, given the sparsity of data on these tumours and their possible malignant potential, which we believe would help potentiate an effective treatment protocol for future cases.

## INTRODUCTION

Parietal peritoneal lipomas are extremely rare in the published literature, and we present a first case report of two parietal peritoneal lipomas incarcerated in an umbilical hernia [1]. The hernia was laparoscopically repaired with a preperitoneal mesh, and the lipomas left *in situ* as they displayed no evidence of torsion or inflammation. We could not clearly ascertain their contribution to the hernial pain.

## CASE REPORT

A 62-year-old gentleman was referred to surgery for a symptomatic umbilical hernia. He had no history of trauma and no previous abdominal wall surgeries. The hernial content was clinically reducible, but the patient did complain of pain on exertion. There were no other abdominal wall herniae.

**Figure 1:**
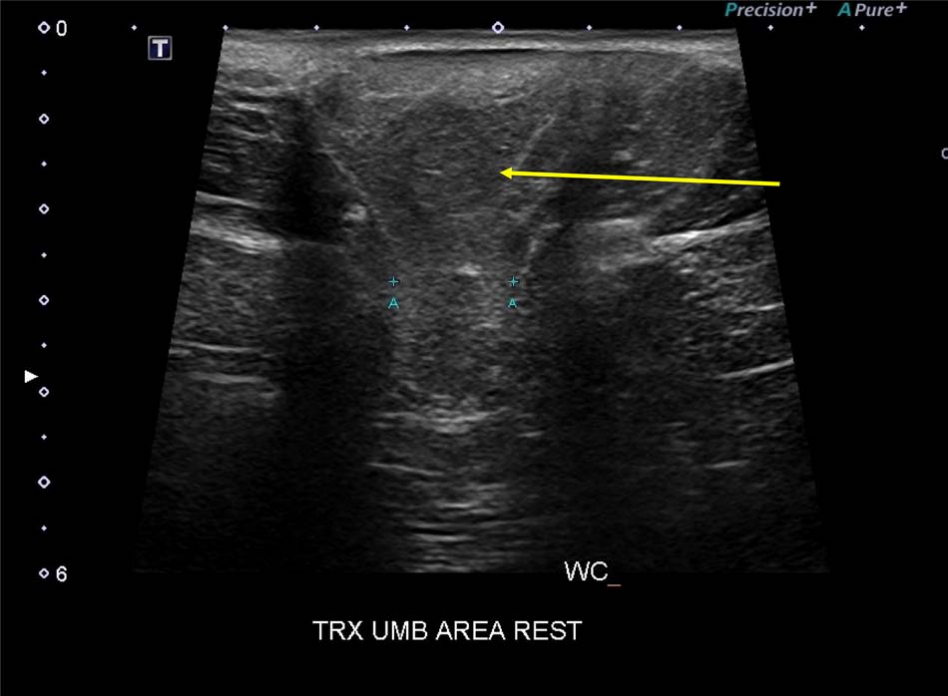
Abdominal wall ultrasound showing the one parietal peritoneal lipoma in the hernia sac (yellow arrow) and the 2 cm hernia neck (A-A).

He did not smoke or lift heavy weights and his medical history was non-contributory. Radiological imaging in the form of an abdominal ultrasound described a fat lesion in the hernia sac (Fig. 1).

After a discussion about the risks and alternatives to surgery, he signed an informed consent for a laparoscopic umbilical herniorrhaphy.

**Figure 2:**
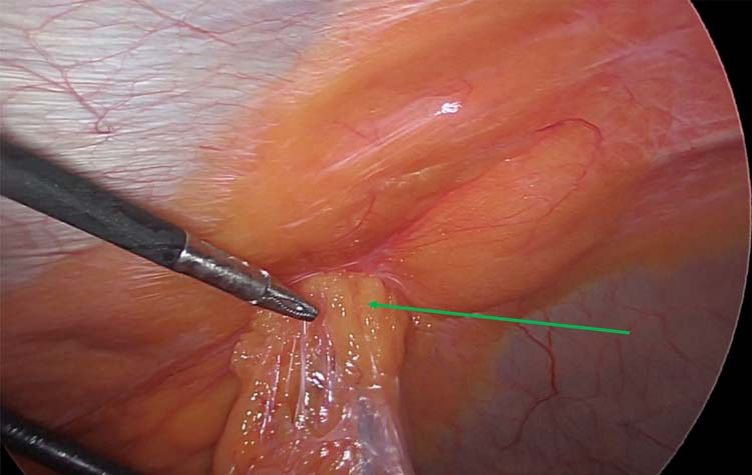
Laparoscopic view of the incarcerated omentum in the umbilical hernia (green arrow).

Intraoperatively, we noticed omentum incarcerated in the hernia sac (Fig. 2).

**Figure 3:**
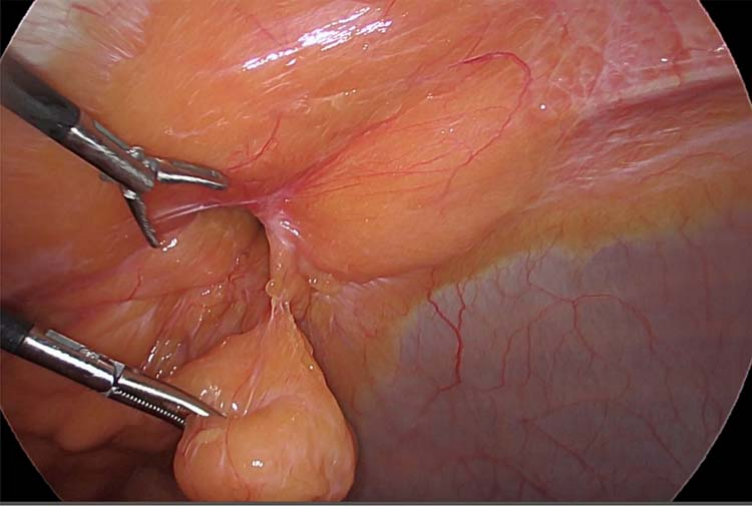
Reduction of the first peritoneal lipoma (green arrow) into the peritoneal cavity.

**Figure 4:**
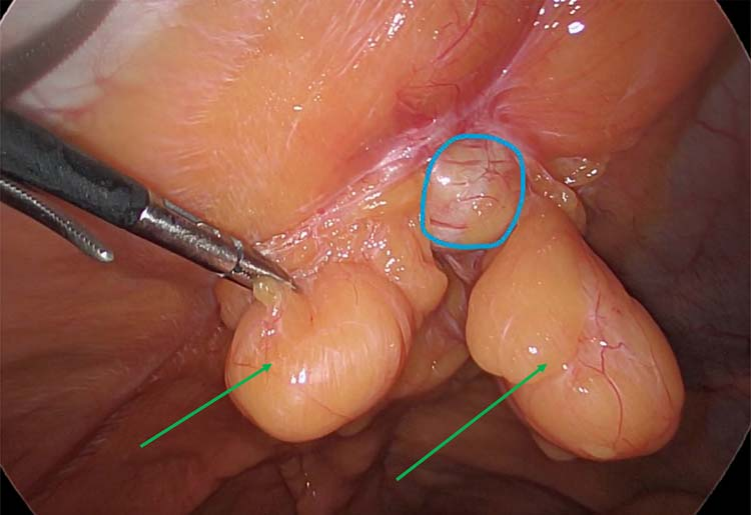
The two peritoneal lipomas (green arrows) and the visible umbilical hernia neck (blue circle). Intracorporeal suturing of the hernia neck (green arrow) and closure of the hernial defect.

The viable omentum was reduced into the peritoneal cavity. Two lipomas were then observed attached to the parietal peritoneum (Figs 3 and 4). The lipomas were also reduced into the peritoneal cavity from within the hernia sac. Each one was 2 cm in diameter based on laparoscopic visualization using the 1 cm markings on a suction irrigation device.

**Figure 5:**
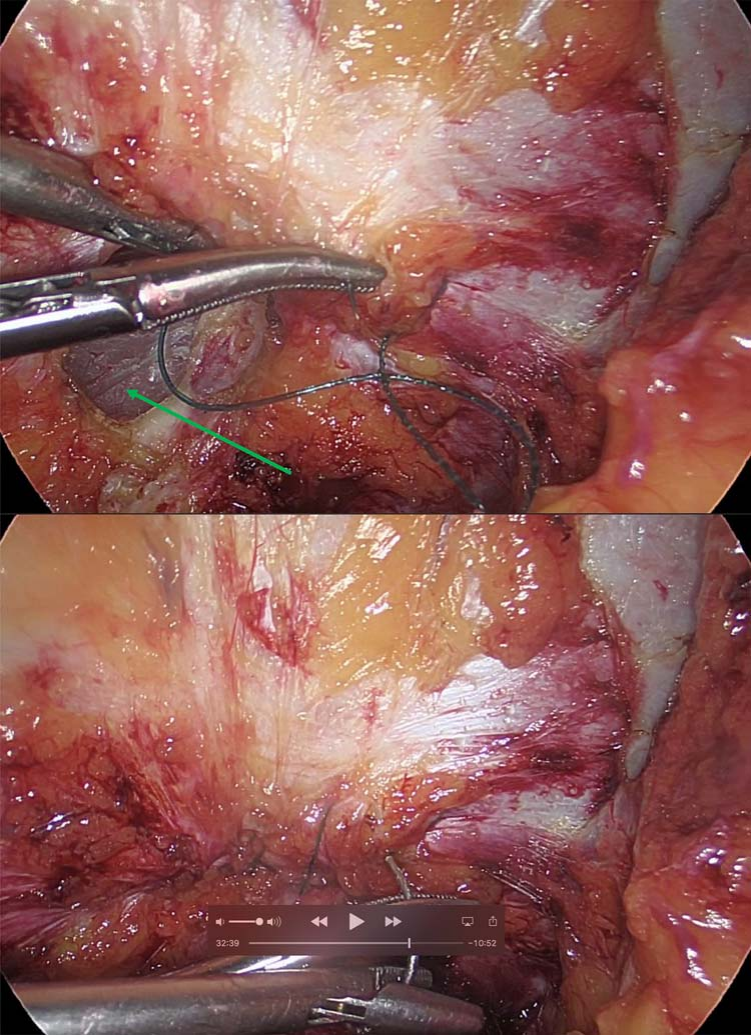
(**a** and **b**) Intracorporeal suturing of the hernia neck (green arrow) and closure of the hernial defect.

**Figure 6:**
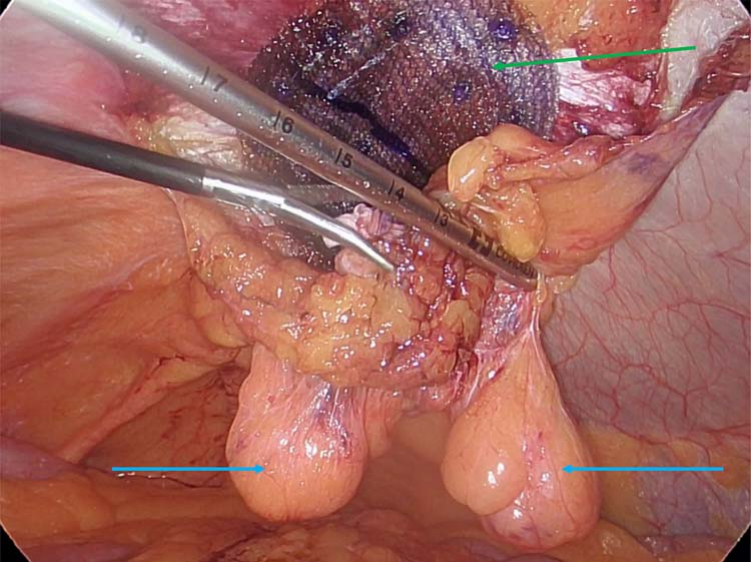
Preperitoneal mesh insertion (green arrow) and visible parietal peritoneal lipomas (blue arrows).

The hernia neck was closed with a 2-0 V-loc^©^ suture (Fig. 5a and b), and a preperitoneal prolene mesh applied. The mesh was secured to the abdominal wall with an absorbable tack fixation device. The mesh was then reperitonealized with absorbable tackers (Fig. 6).

**Figure 7:**
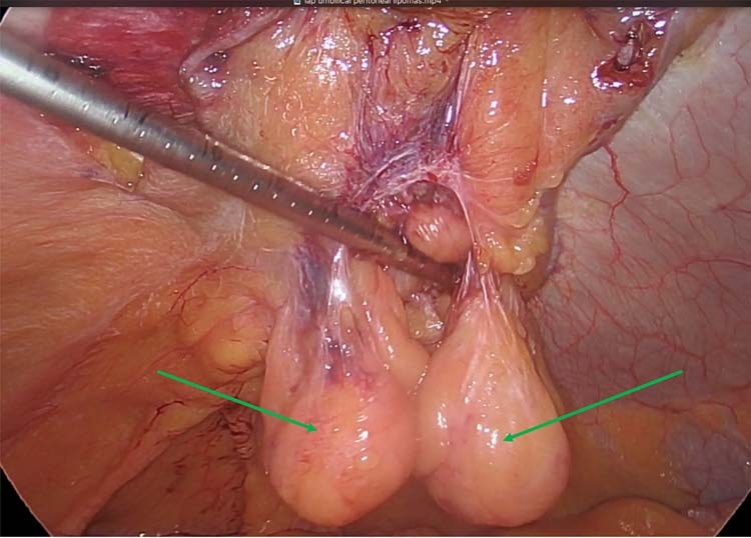
Mesh reperitonealization and parietal peritoneal lipomas *in situ* (green arrows).

The decision was taken intraoperatively to leave the lipomas *in situ* as the hernia neck had been repaired, and it was thought that they could no longer cause any issue with pain or possible hernial reincarceration (Fig. 7).

The patient was discharged home the same day and his recovery has been uneventful.

## DISCUSSION

Parietal peritoneal lipomas are a rare surgical entity with seven documented cases in the published literature since 2006 [1]. This is the eighth report to date and the first reported case with two parietal lipomas that were incidentally discovered during laparoscopic umbilical herniorrhaphy.

The lipomas were incarcerated along with the omentum in the hernia sac, and it was difficult to ascertain whether the lipomas or omentum were responsible for the periumbilical pain in this patient, which necessitated an umbilical herniorrhaphy. The incarcerated omentum did show signs of congestion, which pointed to it as the aetiology of the patient’s periumbilical pain.

The lipomas were not excised as we were uncertain of their contribution to the hernial pain. There were no signs of inflammation or torsion on either lipoma. They reduced easily into the peritoneal cavity.

The hernia neck was initially closed with an absorbable suture and a preperitoneal mesh applied. The mesh was then reperitonealized and the lipomas remained exterior to this. It was felt that the lipomas posed no further risk to incarceration, given the hernial neck closure and application of a preperitoneal mesh, so they were left *in situ*.

Parietal peritoneal lipomas have a low incidence of malignant potential and their excision usually results in a low recurrence rate [2, 3]. They are thought to arise from the mesothelial or submesothelial layers of the peritoneum and their aetiology remains nebulous.

Theories advanced include misplaced embryonic tissue, adipose hyperproliferation, trauma and fat herniation or excessive obesity [3, 4]. In the previously published reports, pain was the resenting feature. There was one case of urinary frequency due to external bladder compression with a large lipoma of 942 g. Management usually involved excision of the lipoma and peritoneum. For the patient with urinary frequency, the lipoma was excised and the peritoneum preserved to reduce the pain associated with a large peritoneal excision [1].

Given the extreme rarity of this tumour, we would advocate for a rare tumour registry so as to collate data of the various cases and guide our future management. Although there have been no documented cases of malignant transformation, it behoves us to have a strategy in place for future reference.

## CONCLUSION

We present the first case report in the published literature of multiple parietal peritoneal lipomas and their conservative management.

## CONFLICT OF INTEREST STATEMENT

None declared.

## FUNDING

None.
